# Calpain Inhibitor PD150606 Attenuates Glutamate Induced Spiral Ganglion Neuron Apoptosis through Apoptosis Inducing Factor Pathway In Vitro

**DOI:** 10.1371/journal.pone.0123130

**Published:** 2015-04-15

**Authors:** Zhong-Jia Ding, Xin Chen, Xiao-Xu Tang, Xi Wang, Yong-Li Song, Xiao-Dong Chen, Wen-Juan Mi, Jian Wang, Ying Lin, Fu-Quan Chen, Jian-Hua Qiu

**Affiliations:** 1 Department of Otolaryngology-Head and Neck Surgery, Xijing Hospital, Fourth Military Medical University, Xi'an, China; 2 Outpatient Department of Logistics Academy, Beijing, China; Massachusetts General Hospital/Harvard Medical School, UNITED STATES

## Abstract

**Objective:**

This research aimed to investigate whether glutamate induced spiral ganglion neurons (SGNs) apoptosis through apoptosis inducing factor (AIF) pathway. And verify whether PD150606, a calpain inhibitor could prevent apoptosis by inhibiting cleaving and releasing AIF in mitochondrion.

**Methods:**

SGNs of postnatal days 0-3 were harvested and cultured in dishes. 20 mM Glu, the caspase inhibitor Z-VAD-FMK and calpain inhibitor PD150606 were added into cultured dishes separately. We used optical microscope and immunofluoresence staining to observe cell morphology and AIF distribution, RT-PCR and Westernblot to analyse AIF and calpain expression in SGNs. TUNEL assay was used to test cell apoptosis.

**Results:**

Cell morphology and nuclear translocation of AIF were altered in SGNs by 20 mM Glu treated in vitro. The axon of SGN shortened, more apoptosis SGN were observed and the expression of AIF and calpain were up-regulated in Glu-treated group than the normal one (P<0.05). The same experiments were conducted in 20 mM+PD150606 treated group and 20 mM+Z-VAD-FMK group. Obviously AIF were located from cytoplasm to the nuclear and the expressions of AIF and calpain were down-regulated by PD150606 (P<0.05). Positive cells in TUNEL staining decreased after PD150606 treating. However, Z-VAD-FMK had no influence on AIF, calpain expression or cell apoptosis.

**Conclusion:**

The AIF-related apoptosis pathway is involved in the process of Glu-induced SGN injury. Furthermore, the inhibition of calpain can prevent AIF from releasing the nuclear or inducing SGN apoptosis.

## Introduction

Apoptosis inducing factor (AIF) plays a key role in the process of cell apoptosis, as an AIF-related apoptosis pathway[1]. From 1994, mitochondrial membrane potential decrease has been discovered in apoptosis[2], and mitochondrion has become the focus of apoptosis research. AIF is a mitochondrial inter-membrane flavo-protein that can be released from mitochondrion to nucleus to induce chromatin condensation and large-scale DNA fragmentation[3,4]. In addition to its binding to DNA, AIF is also a redox enzyme, which plays a role in nicotinamide adenine dinucleotide (NADH) oxidase[5]. The NADH oxidase activity of AIF is separable from its DNA-binding activity to induce apoptosis.

Calpain is a calcium-regulated neutral cysteine protease in cytoplasm and mitochondrion[6]. It could cleave AIF to mature in mitochondrion and release mature AIF through permeability transition pore (PTP) to nucleus inducing cell apoptosis[7]. Calpain could be repressed to cleave AIF by PD150606 binding to the calcium site[3].

In auditory system, apoptosis of sensory cells including SGNs and hair cells plays important role in hearing loss[8]. However, AIF-related apoptosis pathway in sensory cells is yet to be elucidated clearly. Researchers exposed broadband noise or gentamicin to guinea pigs and found AIF translocation to nucleus in outer hair cells[9,10]. A recent report showed that 20 mM Glu perfused into guinea pigs’ inner ears could induce AIF nuclear-translocation in hair cells, which was a homeostatic signal of AIF-related apoptosis pathway[11]. But the questions whether AIF-related apoptosis pathway is associated with Glu-treated SGNs in vitro, and how to inhibit the process still remain open.

## Materials and Methods

### Animals and ethics statement

Twenty postnatal day 1 SD rats were provided by and cared for in the Institutional Animal Care and Use Facility of the Fourth Military Medical University in Xi’an, China. Rats were decollated and SGNs were dissociated under sterile conditions excluding the stria vascularis, the spiral ligament and the basilar membrane. The study was approved by Xijing Hospital’s ethics committee.

### Spiral ganglion neuron culture

The SGNs were incubated in Dulbecco’s modified Eagle’s medium supplemented (B272 ml/ml Sigma USA; BDNF 10 μg/ml Sigma USA, penicillin 100000 U/L 1% Sigma USA) at 37°C in a humidified incubator with 5% CO_2_[12]. After 24 h incubation, culture dish of SGNs were divided into four dishes for different interventions. 20 mM Glu, 20 mM+PD150606 and 20 mM+Z-VAD were added to different dish as drug instruction. Then we observed and disposed after 48 h cells incubation.

### Immunofluorescence staining

Slips of SGNs were fixed in 4% paraformaldehyde 30 min and permeabilized in 0.1% TrionX-100 15 min. After washing by PBS, they were incubated in blocking solution of bovine serum albumin (BSA, 5%, Sigma, USA) 20 min and in antibodies against AIF (1:200, rabbit, Abcam, USA) and β-tubulin (1:200, mouse, Abcam, USA) 24 h overnight at 4°C. Alexa-488 conjugated goat anti rabbit (1:200, Invitrogen, USA) and Alexa-594 conjugated donkey anti mouse (1:200, Invitrogen, USA) were used to label the primary antibodies for incubation 40 min at 37°C. Staining SGNs were observed under a fluorescence microscope (Olympus, Japan) and three photos of different groups were taken by microscope.

### TUNEL assay

A TUNEL kit (Roche red TUNEL kit, Germany) was used to detect DNA fragmentation at 48 h after different interventions. Cells were stained following TUNEL staining instructions. Three pictures were taken from each group by microscope. Two independent and blinded individuals counted the cultured SGNs that were positive or negative in the TUNEL staining pictures.

### Real-time quantitative PCR

The RNAs were isolated by RNA extraction kit (Qiagen, Germantown, MD, USA). The genomic DNAs were composed according to the QuantiTect Reverse Transcription Handbook (Qiagen, Germantown, MD, USA). Quantitative PCR analysis was performed using a SYBR Green Master Mix Kit (Applied Biosystems, Foster City, CA, USA) and RNase-free 96-well PCR plates. The following forward and reverse primers (GeneCopoeia, USA) were used for the specific RNAs in the quantitative PCR experiments:

- R-aif-F: 5' TAGAACTCCAGATGGCAAGACA 3';
- R-aif-R: 5'AAGCCCACAATAAGGACTAACAC3';
- R-caspase3-F: 5'GAATGACTGGGAGTGGGGTAG 3';
- R-caspase3-R: 5' GACCTGGAACATCGGATTTGA 3';
- R-calpain-F: 5' CAAAGTGGACCCCTATGAACG 3';
- R-calpain-R: 5' TAAGGGCGTCAGGTGTA AGGT 3'.

The positive and negative control reactions were also performed in triplicate. Each sample exhibited a particular threshold cycle (Ct) value during amplification, which was a representative of the relative abundance of the mRNA in the sample RNA. The relative fold change in gene expression was obtained by a comparison of the 2^-ΔΔCt^ data of the different groups.

### Western blot analysis

Cells were washed with PBS and lysed in ice-cold RIPA buffer (10 mM Tris-HCl, 1 mM EDTA, 1% sodium dodecyl sulfate, 1% Nonidet P-40, 1:100 proteinase inhibitor cocktail, 50 mM β-glycerophosphate, and 50 mM sodium fluoride). The BCA Protein Assay Kit (Beyotime, Shanghai, China) was used to detect the protein content of the lysates according to the manufacturer’s protocol. The proteins were loaded on 10% sodium dodecyl sulfate polyacrylamide gels (SDS-PAGE), transferred to polyvinylidene fluoride (PVDF) membranes (Bio-Rad, Hercules, CA, USA) and blocked with 5% nonfat milk powder in tris buffered saline tween (TBST). The membranes were incubated overnight with the following monoclonal primary antibodies: AIF (1:1000, rabbit, Abcam, USA), calpain (1:1000, mouse, Abcam, USA), active-caspase-3 (1:1000, rabbit, Abcam, USA), and β-actin (Santa Cruz Biotechnology, USA, 1:4000). The membranes were incubated with secondary conjugated horseradish peroxidase antibodies (Beyotime) for 2 h at room temperature. The blots were visualized using an enhanced chemiluminescence kit (Amersham Biosciences, Piscataway, NJ, USA).The band intensities were obtained by a gel imaging system (Syngene, USA) and intensities were quantified with the software of image J.

### Data analysis

Data was collected from three experiments independently. Statistical significance was determined using analysis of variance (ANOVA) followed by a multiple comparison Dunnett's test (using SPSS software). A P value of less than 0.05 was considered significant.

## Results

### Glu shortened SGN axons and induced SGN apoptosis

**Fig 1:**
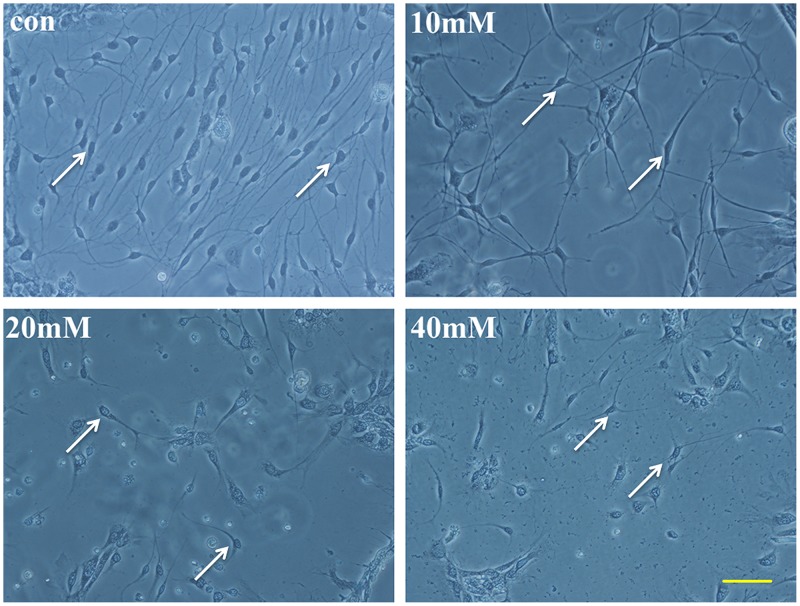
The morphology of SGNs in different interventions in stereoscopic microscope. Glu and two inhibitors were added to the SGNs in integrated medium. In addition to the control group, the drugs that were added to the cultured SGNs included 20 mM Glu, 20+PD150606 and 20+Z-VAD-FMK. Morphological changes were clearly observed in the four groups. The neurites of the SGNs were counted by Image-Pro Plus IPP with the scales. Scale bar = 100 μm.

Morphological changes of SGN occurred after 20 mM Glu adding to the dishes for 48 h. The neurite extension was detected by the software IPP and scale label. The average length of the longest five neurites was calculated. We detected that the average neurite extension of the 20 mM Glu-treated group was 52.2±4.3 μm, which was significantly shorter than the normal group’s value of 114.8±7.3 μm. The neurites shortened and vacuoles occurred in the cytoplasm of SGNs following 20 mM Glu treating for 48 h (Fig 1). PD150606 shortened SGN neuritis of the value of 64.3±14.7μm in 20 mM Glu+PD150606-treated group. However, the extension of Z-VAD-FMK-treated group was longer than PD150606-treated group of 83.1±24.3μm.

**Fig 2:**
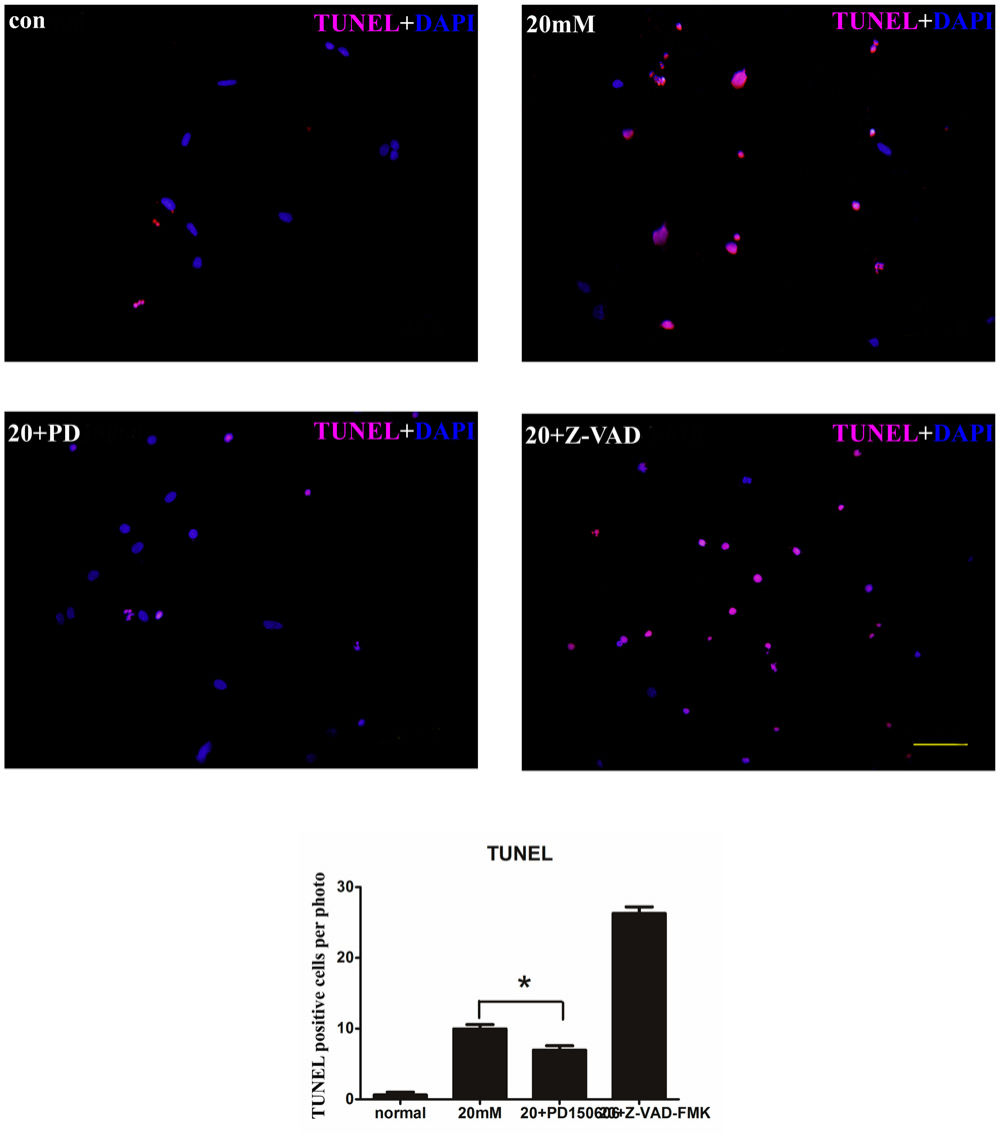
TUNEL staining of SGNs in different interventions. TUNEL staining in cultured SGNs. Red overlapped with the blue nuclei indicates a positive cell. (A) The cells in the normal condition. (B) The cells that were treated with 20 mM Glu. (C) The cells that were treated with 20 mM Glu and PD150606. (D) The cells that were treated with 20 mM Glu and Z-VAD-FMK. Photos were taken under the fluorescence microscope. Scale bar = 50 μm. Positive cells were counted in each photo and a histogram was drawn. The cell count for 20 mM Glu+PD150606-treated group was lower compared with 20 mM Glu-treated group (P<0.05).

We also used TUNEL staining assay to perform apoptosis of cultured SGNs. After 48 h incubation of 20 mM Glu treating, a greater number of TUNEL positive apoptotic cells could be identified in the photographs (P<0.05). An average of 9.3±2.1 apoptotic cells were counted in the three pictures in 20 mM, while 1±0.6 was counted in the control group (Fig 2).

### Toxicitic Glu induced AIF nuclear translocation and AIF, calpain up-regulation in cultured SGNs

**Fig 3:**
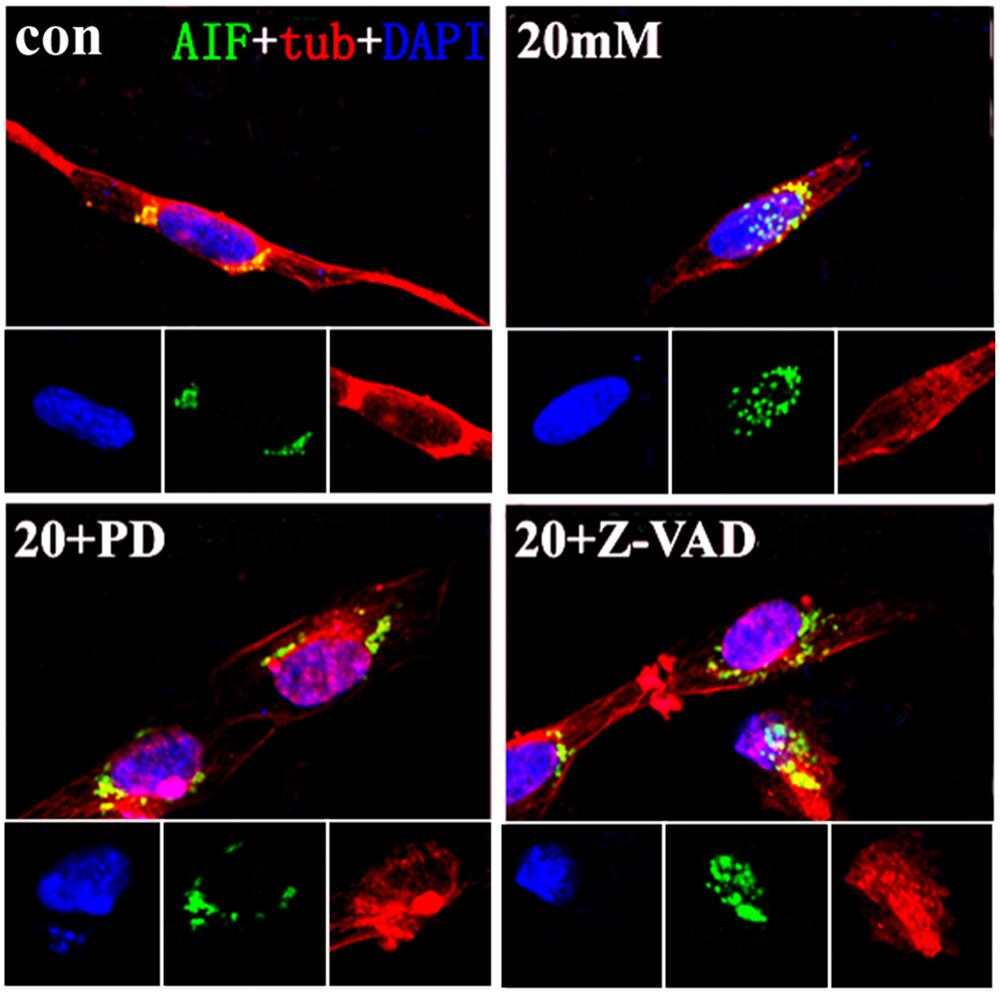
Immunofluorescence staining of SGNs in different interventions. Immunofluorescence staining in cultured SGNs. (A) (B) (C) (D) were treated as previously described in Fig 2. The AIF staining was distributed in the cytoplasm of the SGNs in the normal condition. Clear translocation into the nuclei of AIF was observed in the 20 mM Glu-treated and 20 mM Glu+Z-VAD-FMK-treated groups. No translocation was observed in the 20 mM Glu+PD150606-treated group. Scale bar = 20 μm.

**Fig 4:**
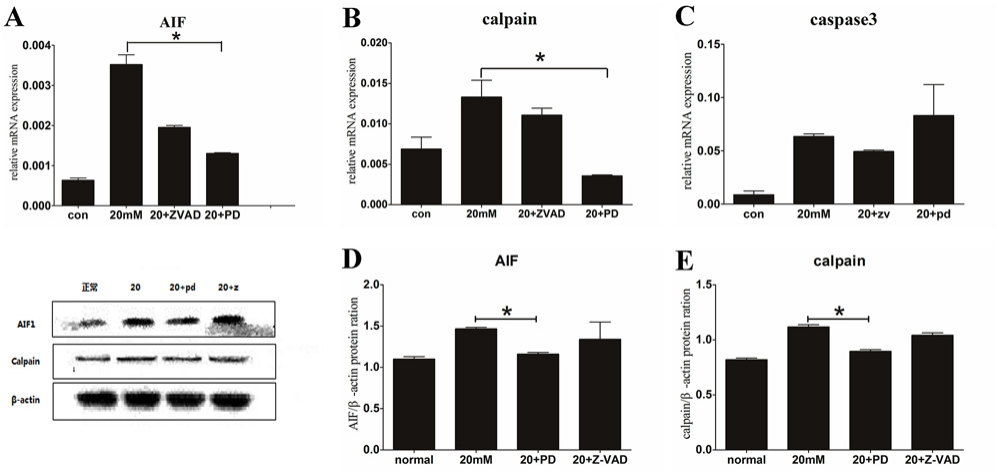
Relative expression of AIF and calpain in RT-PCR and WB. A, B, C show the SGN mRNA expression of AIF, calpain and caspase-3 in RT-PCR, respectively. The 20 mM Glu+PD150606-treated group showed a significant decrease compared with the 20 mM Glu-treated in AIF and calpain expression (P<0.05); however, there was no significant difference in caspase-3 expression (P>0.05). The three photos below show the western blot results of the cultured SGNs. D, E represent the protein ration of AIF and calpain in all groups, respectively. The 20 mM Glu+PD150606-treated group had a minimal decrease compared with the 20 mM Glu-treated group for both AIF and calpain expression, which was consistent with the RT-PCR results.

Immunofluorescence staining was used to present AIF distribution in different groups. Three pictures were taken and processed by the fluorescence microscope and Microsoft fireworks. The most clearly pictures were selected and showed that AIF was located in cytoplasm and absent from the nucleus in normal group. However, AIF staining tranlocated to nucleus obviously by 20 mM Glu-treated (Fig 3).That was a homeostatic signal of AIF-related apoptosis, distinguished from its role inmitochondrion. Calpain expression changed in 20 mM Glu-treated group, which was in agreement with AIF expression by RT-PCR. Up-regulation in AIF and calpain’s mRNA were showed after 20 mM Glu treating than the normal group (P<0.05) (Fig 4A and 4B). The western blot results to some degree supported the up-expression of AIF and calpain. An 80kDa protein bind of calpain had an increased grey level in 20 mM Glu-treated group, and the grey level of AIF bind increased too (Fig 4D and 4E). The increased expressions of calpain and AIF indicated that AIF played a critical role in apoptosis of SGNs in vitro, which was similar to apoptosis of HT22 cells induced by Glu.

### PD150606 inhibited AIF, calpain up-regulation and cell apoptosis.

(2S)-3-(4-iodophenyl)-2-sulfanylpropanoic acid (PD150606) was an inhibitor of calpain, it could block calpain bonding sites of Ca^2+^. We added PD150606 to the cultured dishes 1 h prior to the Glu intervention, as had been previously described. Following the 48 h of Glu intervention, AIF was located in the cytoplasm, but not in the nucleus. That was different from AIF nuclear-translocation in 20 mM Glu-treated and 20 mM Glu+Z-VAD-treated groups (Fig 3).The TUNEL apoptosis staining showed a decrease in the amount of positive cells by PD150606-treated than 20 mM Glu-treated group (Fig 2B and 2C), and the expression of AIF declined when we used PD150606 to inhibit calpain. Down-expression of AIF by RT-PCR was showed in PD150606-treated group than 20 mM Glu-treated group obviously (P<0.05), and the mRNA of calpain was performed similar results (P<0.05) (Fig 4A and 4B). We also used western blot to investigate the expression of AIF and calpain in SGN, PD150606 could attenuate up-expression of protein AIF (67 kDa) and calpain (80 kDa) (Fig 1). That was consistent with the AIF and calpain mRNA translation in RT-PCR results (Fig 4D and 4E). In conclusion, PD150606 could attenuate SGN apoptosis induced by Glu in AIF-related manner.

### Z-VAD-FMK has no effect on apoptotic cells of cultured SGNs induced by Glu

To study caspase-3 in apoptosis of cultured SGNs induced by 20 mM Glu, Z-VAD-FMK, the caspase inhibitor, was added to cultured dishes as previously described. Apoptotic cell counting, RT-PCR, and western blot were performed in Z-VAD-FMK treating dish. The number of TUNEL positive cells was greater in the Z-VAD-FMK-treated group than that in 20 mM Glu-treated group (P<0.05), but a decline was observed in 20 mM+PD150606-treated group than the 20 mM Glu-treated group (Fig 2). ThePCR histograms presented that, the mRNA of caspase-3 does not increase in 20 mM Glu-treated group compared with the other groups (Fig 4C). Western blot also showed no expression of the activecaspase-3, data not shown. Thus, these findings indicate that inhibition of the caspase-dependent pathway has no effective influence on cultured SGN apoptosis induced by 20 mM Glu.

## Discussion

The dysfunction and injury of SGNs in cochlea were important causes to hearing loss. The mechanism may be cell apoptosis induced by pathological factors in SGNs[13]. In our research, 20 mM Glu was used to damage SGNs in vitro, the axons of SGN shortened and AIF was located into cell nuclear in morphology changes, which was a homeostatic signal in AIF-related apoptosis[6]. The expressions of AIF and calpain were up-regulated by Glu toxicity in SGNs[14,15]. After PD150606 treating, positive results were showed like that cell morphologe altered, AIF located outside the cell nucleus and AIF, calpain were down-expression. However, Z-VAD-FMK didn’t have the same effect.

In dead cells, AIF could be released from the inter-membrane space of the mitochondrion, translocated into cytosol and subsequently to nucleus where to induce chromatin condensation and large scale DNA fragmentation in apoptosis[16]. But what could regulate AIF releasing was still unknown[17]. A recent research showed calpain cleavage and up-expression of AIF after stimuli treating in HT22 cells apoptosis[18]. In cell-free system, calpain was proved to cleave AIF to mature and promote AIF releasing[19]. In this study, we obtained positive results that calpain and AIF were up-expressed and AIF was nuclear-translocated. Furthermore, AIF up-regulation also observed in cochlea sensory cells apoptosis induced by Glu, which was in agreement with the AIF-expression in our results[20]. So AIF was associated with apoptosis cells induced by Glu. Meanwhile calpain up-expression was also detected. In conclusion, calpain could promote AIF nuclear-translocation and AIF up-expression in Glu-induced cells.

Recently, authors in auditory research regarded caspases as important apoptosic molecules in sensory cells of inner ear[19]. The inhibition of caspases (capase-3, capase-8) resulted in incomplete or limited neuronal protection, such as attenuating neuritis shorten in my article. These results suggest that additional pathways of neural cell death might exist[21]. We drew this conclusion by inhibition of caspase-3. A possible mechanism is that the exposure to Glu might induce a translocation of AIF to the nucleus where it likely acts to induce apoptosis in a caspase-independent manner but not caspase-dependent manner.

It was reported that AIF nuclear-translocation occurred in a number of cell types, independent of the nature of the death-triggering signal[22]. However, whether it occurred in other sensory cells remained to be answered in our future works. The inhibition of calpain was involved in apoptotic SGN decrease[23] and AIF outside the nuclear in our results. Although morphology of SGNs was not rescued completely, the inhibition of calpain could prevent to a great degree Glu-induced SGNs apoptosis in AIF-related manner.

In conclusion, the present study demonstrated that AIF participates in Glu-induced apoptosis of SGNs in vitro. Calpain is involved in AIF nuclear-translocation and AIF up-expression. Inhibition of calpain contributes to AIF outside to nuclear and AIF down-expression. The precise mechanism of AIF-related apoptosis needs to be further explored. And a new explain could be offered for treatment of hearing loss.
